# Genetic alterations and functional networks of m6A RNA methylation regulators in pancreatic cancer based on data mining

**DOI:** 10.1186/s12967-021-03001-2

**Published:** 2021-07-30

**Authors:** Juan Zeng, Heying Zhang, Yonggang Tan, Zhe Wang, Yunwei Li, Xianghong Yang

**Affiliations:** 1 Department of Oncology, Shengjing Hospital of China Medical University, Shenyang, 110004 Liaoning People’s Republic of China; 2 Department of Pathology, Shengjing Hospital of China Medical University, Building 1, No.36, Sanhao Street, Heping District, Shenyang, 110004 Liaoning People’s Republic of China

**Keywords:** N6-methyladenosine (m6A), m6A RNA methylation regulators, Pancreatic cancer, Genetic alterations, Survival

## Abstract

**Background:**

Pancreatic cancer is a fatal malignancy of the digestive system and the 5-year survival rate remains low. Therefore, new molecular therapeutic targets are required to improve treatments, prognosis, and the survival of patients. N6-methyladenosine (m6A) is the most prevalent reversible methylation in mammalian messenger RNA (mRNA) and has critical roles in the tumorigenesis and metastasis of various malignancies. However, the role of m6A in pancreatic cancer is still unclear. Exploring genetic alterations and functional networks of m6A regulators in pancreatic cancer may provide new strategies for its treatment.

**Methods:**

In this study, we used data from the Cancer Genome Atlas (TCGA) database and other public databases through cBioPortal, LinkedOmics, UALCAN, GEPIA, STRING, and the database for annotation, visualization, and integrated discovery (DAVID) to systematically analyze the molecular alterations and functions of 20 main m6A regulators in pancreatic cancer.

**Results:**

We found that m6A regulators had widespread genetic alterations, and that their expression levels were significantly correlated with pancreatic cancer malignancy. Moreover, m6A regulators were associated with the prognosis of pancreatic cancer patients.

**Conclusions:**

m6A regulators play a crucial part in the occurrence and development of pancreatic cancer. Our study will guide further studies of m6A RNA modification in pancreatic cancer and could potentially provide new strategies for pancreatic cancer treatment.

**Supplementary Information:**

The online version contains supplementary material available at 10.1186/s12967-021-03001-2.

## Background

Pancreatic cancer is a fatal malignancy of the digestive system, the fourth leading cause of cancer-related deaths in the United States, and the seventh most common cause of cancer-related deaths worldwide [1, 2]. The median survival period for pancreatic cancer is 3–6 months. Despite medical advancement, the 5-year survival rate for pancreatic cancer remains the lowest among all tumors (9% in the United States from 2009–2015 [1], < 5% worldwide [3]). Although improvements in early detection, surgical techniques, and adjuvant medical therapy have led to a sustained decline in the overall mortality rate of cancer, the death rate of pancreatic cancer has risen over the past decade [1]. In China, the incidence and death rate for pancreatic cancer are both increasing. According to Global cancer statistics 2012, 19.45% of all newly diagnosed pancreatic cancer cases and 19.27% of all pancreatic cancer-related deaths worldwide occur in China. Estimates indicate that in 2035, approximately 130,365 new patients in China will be diagnosed with pancreatic cancer, and nearly 130,000 new deaths will occur annually [4]. As pancreatic cancer is insidious and often asymptomatic before metastasis, more than 80% of patients presents at a late stage at first diagnosis. Moreover, in most cases, lesions cannot be completely removed by surgery, postoperative recurrence and mortality rates are high, and prognosis is poor [2]. Therefore, the molecular mechanisms of the occurrence and development of pancreatic cancer must be studied. Further, we must identify new molecular therapeutic targets and improve treatments, prognosis, and the survival of pancreatic cancer patients.

Pancreatic cancer tumorigenesis and metastasis are multifactorial and multistage processes involving genomic changes and epigenetic disorders [5, 6]. As a key element in the Central Dogma of Genomics, RNA binds DNA to proteins, which carry out vital movement. However, the number and abundance of proteins expressed in cells are often not equivalent to those for mature mRNA. This indicates that post-transcriptional modification plays an important regulatory role in basic living processes. MODOMICS announced that more than 160 different chemical modifications have been identified in RNA molecules so far, including m6A, 5-methylcytosine, *n*7-methylguanosine, *n*1-methyladenosine, and pseudouridine [7].

m6A is the most important modification occurring in eukaryotic mRNA and long noncoding RNA (lncRNA) [8]. In 1974, Desrosiers et al. first discovered m6A in the purified poly (A) RNA fraction of Novikoff hepatoma cells [9]. Similar to DNA or protein methylation, m6A is reversibly regulated by different types of regulators, including “writers,” “erasers,” and “readers.” m6A methyltransferases such as methyltransferase-like (METTL3) 3, METTL14, Wilms tumor 1-associated protein (WTAP), RNA-binding protein 15/15B (RBM15/15B), vir like m6A methyltransferase associated protein (VIRMA), and zinc finger CCCH domain-containing protein 13 (ZC3H13) work as “writers,” and can catalyze m6A formation. m6A demethylases such as fat-mass-and obesity-associated protein (FTO) and its homolog AlkB family member 5 (ALKBH5) are “erasers,” and selectively remove the methylated modification from target RNAs. m6A can regulate multiple steps of the RNA life cycle when it is recognized by m6A-specific binding proteins (denoted as “readers”), including YT521-B homology (YTH) domain-containing protein, insulin-like growth factor 2 mRNA-binding protein 1/2/3 (IGF2BP1/2/3), eukaryotic translation initiation factor 3 subunit A (EIF3A), and heterogeneous nuclear ribonucleoproteins A2/B1 (HNRNPA2B1).

m6A is involved in almost every step of the RNA life cycle, including mRNA transcription, splicing, nuclear export, localization, translation, stability, and degradation [10]. A growing body of evidence has suggested that m6A has vital roles in cancer development and progression [11, 12]. However, it is still unknown how these different m6A regulators are altered in pancreatic cancer, in addition to how dysregulation of m6A modification mediated by these regulators affects cell proliferation and apoptosis, thus leading to the development of pancreatic cancer.

In this study, we conducted bioinformatics analysis of public sequencing data and found that m6A regulator expression had widespread alterations in pancreatic cancer. Moreover, Kaplan–Meier survival analysis showed that the expression of m6A regulators was associated with overall survival (OS) and disease/progression-free survival (DFS/PFS) in pancreatic cancer patients. These results suggest that m6A regulators may be useful prognostic markers for pancreatic cancer. Thus, we studied the expression and mutations of 20 main m6A regulators (eight m6A writers: *METTL3*, *METTL14*, *WTAP*, *RBMX*, *RBM15*, *RBM15B*, *VIRMA*, and *ZC3H13*; two m6A erasers: *FTO* and *ALKBH5*; and ten m6A readers: *YTHDF1*, *YTHDF2*, *YTHDF3*, *YTHDC1*, *YTHDC2*, *HNRNPA2B1*, *EIF3A*, *IGF2BP1*, *IGF2BP2*, and *IGF2BP3*) in data from patients with pancreatic cancer in TCGA and various public databases [13]. We systematically characterized genomic alterations and functional networks related to m6A regulators in pancreatic cancer. Our study highlights the importance of m6A regulators in pancreatic cancer development, and could potentially provide new targets and strategies for its diagnosis and treatment.

## Methods

### Bioinformatics databases and analysis

#### cBioPortal analysis

The cBioPortal for Cancer Genomics (http://cbioportal.org) is an open web-based platform for exploring, visualizing, and analyzing multidimensional cancer genomics data [14]. cBioPortal significantly reduces the barriers to access of complex genomic data to cancer researchers, promotes rapid, intuitive, and high-quality access to molecular profiles and clinical prognostic correlations from large-scale cancer genomics projects, and enables researchers to translate these rich data sets into biological insights and clinical applications. We initially used the cBioPortal to analyze the genetic alterations in 20 main m6A regulators in 776 cases from 4 pancreatic cancer studies. We explored amplification, mutation, deep deletion mutation, copy number alterations (CNAs), and mRNA expression. The tab OncoPrint showed an overview of the genetic alterations present in the 20 main m6A regulators in each case. We then investigated the crosstalk among m6A writers, readers, and erasers in pancreatic cancer. We also evaluated the survival data and investigated the effect of m6A regulators alterations on the DFS/PFS and OS of pancreatic cancer patients.

#### GEPIA

Gene Expression Profiling Interactive Analysis (GEPIA) database integrates TCGA data and Genotype-Tissue Expression (GTEx) data, and uses bioinformatics technology to reveal cancer subtypes, driver genes, alleles, differential expression or carcinogenic factors, so as to dig deep into new cancer targets and markers [15]. In general, GEPIA database integrates current cancer genomics data, which can be used to mine data more easily and quickly, and to conduct dynamic analysis of gene expression profile data. We used GEPIA to explore the mRNA expression of m6A regulators between pancreatic adenocarcinoma (PAAD) and normal samples. GEPIA is available at http://gepia.cancer-pku.cn/.

#### UALCAN analysis

UALCAN is a comprehensive, user-friendly, interactive web resource for analyzing TCGA data [16]. UALCAN analysis serves as a platform to validate target genes in computer simulations and identify candidate biomarkers specific to tumor subpopulations. Therefore, the UALCAN portal is very helpful in accelerating cancer research. We used UALCAN to analyze multiple clinicopathological characteristics of 178 PAAD samples and 4 normal samples in TCGA. UALCAN is publicly available at http://ualcan.path.uab.edu.

#### LinkedOmics analysis

LinkedOmics is a new portal for analyzing multi-omics data and clinical data from TCGA [17]. The whole analysis process needed to submit a query consists of five steps: (1) Select tumor types (there are 32 cancer types included), (2) Select data types, including microRNAs, single nucleotide polymorphisms (SNPs), methylation, clinical data, and mutations, (3) Select data attributes, (4) Select the target data type, and (5) Select statistical methods. The analysis results will show the relationship between the target gene and clinicopathological information, including survival data, TNM staging, and ethnicity. You can also obtain gene heat maps of genes positively and negatively correlated with target gene expression. We used LinkedOmics to analyze the mRNA sequencing data of 20 main m6A regulators from 178 PAAD patients in TCGA. Statistical analysis was conducted using Spearman test. The LinkFinder also created statistical plots for individual genes. The coexpression results of ALKBH5 in pancreatic cancer were graphically presented in volcano plots and heat maps. The top 50 genes that were significantly positively and negatively correlated with m6A regulators in pancreatic cancer were used to perform enrichment analyses of Gene Ontology (GO) and Kyoto Encyclopedia of Genes and Genomes (KEGG) pathways. LinkedOmics is freely available at http://www.linkedomics.org.

#### STRING analysis

STRING is an online search database for known protein–protein interaction (PPI) relationships, storing information on 1,380,838,440 interactions of 2,031 species and 9,643,763 proteins [18, 19]. Entering a single protein will show a network composed of all the proteins that interact with the target protein. This function is more suitable for exploring the interaction of a particular protein. Entering multiple proteins will only provide the interaction network between the input proteins, which is more suitable for mining interaction between the input proteins, such as identifying all the differentially expressed genes from the input transcriptome data, and analyzing the interaction between these genes. We used STRING to explore PPI networks between these writers, erasers, and readers in humans. Significant GO term analysis and Reactome pathway analysis were shown in bar plots. STRING can be freely accessed at https://string-db.org/.

#### DAVID analysis

The database for annotation, visualization, and integrated discovery (DAVID) is a biological information database that integrates biological data and analysis tools to provide systematic and comprehensive annotated biological function information for the large-scale gene or protein lists (hundreds or thousands of gene IDs or protein ID lists) to help users extract biological information from them [20]. We used DAVID to analyze significantly enriched GO terms and KEGG pathways of the top 50 genes significantly positively and negatively correlated with m6A regulators. DAVID is freely available at http://david.abcc.ncifcrf.gov/.

#### Cytoscape

Cytoscape is an open software platform for visualizing complex molecular networks and integrating networks with any type of attribute data [21]. Cytoscape is not only a tool for biological research, but also a general platform for complex network analysis and visualization. Beside, a lot of applications are available for various kinds of problem domains, including bioinformatics, social network analysis, and semantic web. We used Cytoscape to perform the crosstalk among m6A writers, readers, and erasers in humans. In addition, we have visualized the functional enrichment analysis of 20 main m6A regulators in humans. You can load Cytoscape software at http://www.cytoscape.org/download.php.

### Validation of expression level and function of m6A regulators in pancreatic cancer in vitro

#### Cell lines, reagents and culture conditions

Human pancreatic cancer (PC) cell lines AsPC-1(ATCC CRL-1682), BxPC-3 (ATCC CRL-1687), Capan-2 (ATCC HTB-80), Panc-1(ATCC CRL-1469), and SW1990 (ATCC CRL-2172) were purchased from the American Type Culture Collection (ATCC, Manassas, USA). Human immortalized pancreatic duct epithelial cell line HPDE6-C7 was purchased from Biotechnology Company. All cell lines were authenticated by short tandem repeat profiling before receipt, tested for free from mycoplasma infection. AsPC-1, BXPC-3, and HPDE6-C7 cells were maintained in RPMI-1640 medium. Panc-1 was cultured in DMEM medium. SW1990 cell was cultured in L-15 medium. Capan-2 cell was cultured in ATCC-formulated McCoy's 5a Medium Modified (Catalog No. 30-2007). All the mediums were supplemented with 10% fetal bovine serum (FBS) and 1% penicillin/streptomycin. All cell lines except SW1990 cell were grown in an atmosphere of 5% CO_2_ and 99% relative humidity at 37 °C. SW1990 cell was grown in an atmosphere without CO_2_.

#### Antibodies, short hairpin RNAs (shRNAs) and plasmids transfection

The primary antibodies were used to detect protein expression levels of m6A regulators, and purchased from commercial sources, and information about them was given in Additional file 1. METTL14-shRNAs and METTL14 overexpression (METTL14-OE) plasmid were used to knockdown and up-regulate METTL14, respectively, and purchased from Genechem (Shanghai, China). BxPC-3, SW1990 and HPDE6-C7 cells were seeded into 6-well plates at 30–50% density. After 24 h of culture, shMETTL14-09, shMETTL14-10, shMETTL14-11, shRNA negative control (NC), METTL14-OE plasmid, or empty vector plasmid were transfected into BxPC-3, SW1990 and HPDE6-C7 cells, respectively, using jetPRIME agent (Polyplus Transfection, Illkirsch, France) according to the manufacturer’s instructions. 16 h after transfection, cells were seeded into 96-well plates.

#### RNA isolation and reverse transcription

Total RNA was isolated from AsPC-1, BxPC-3, Capan-2, Panc-1, SW1990, and HPDE6-C7 cells by using RNAiso Plus reagent (TaKaRa, Beijing, China) according to the manufacturer’s instructions. Synergy ™ H1 microplate reader (BioTek Instruments Inc., Winooski, USA) was used to determine RNA purity and to quantify RNA concentration. Reverse Transcription Kit (Takara, Beijing, China) was used to perform reverse transcription.

#### Real-time quantitative polymerase chain reaction (RT-qPCR)

RT-qPCR was used to assess the relative abundance of mRNA. RT-qPCR primers were obtained from Sangon Biotech (Shanghai, China), and the sequences were listed in Additional file 1. According to the manufacturer’s instructions, RT-qPCR was performed through using the SYBR Prime-Script RT-PCR Kit (Takara, Beijing, China) with a 7500 Fast Real-Time PCR System (Thermofisher Scientific, New York, USA). The reaction started at 95 °C for 30 s, followed by 40 cycles of 95 °C for 5 s, 60 °C for 34 s, then entered the dissociation stage (95 °C for 15 s, 60 °C for 1 min, and 95 °C for 15 s). GAPDH (for mRNA expression level) was used as an internal control to normalize qPCR results. Relative gene expression levels were measured using cycle threshold (CT) values in the ΔΔCT calculation. Each experiment was performed in triplicates and repeated three times.

#### Western blotting (WB) analysis

WB analysis was performed to detect relative protein expression levels of m6A regulators. Briefly, cells were washed with ice-cold phosphate buffer saline three times and lysed in RIPA lysis (Beyotime, Beijing, China) supplemented with a protease inhibitor cocktail (Roche, Shanghai, China) and PMSF (Roche, Shanghai, China) at 4 °C for 30 min. Then, samples were centrifuged at 12,000 rpm at 4 °C for 30 min in a low-temperature refrigerated centrifuge, and the supernatants were retained as protein lysates. The protein lysates were measured and determined by utilizing BCA protein assay kit (Beyotime, Beijing, China). The protein lysates were mixed with 1/4 volume of 5 × sodium dodecyl sulfate sample buffer and boiled for 10 min. For immunoblotting, 60 μg of protein lysates were separated by 10% sodium dodecyl sulfate-polyacrylamide gel electrophoresis; transferred to polyvinylidene difluoride membranes (Solarbio; Beijing, China); and then blocked with 5% (w/v) skim milk in Tris-buffered saline-Tween 20 (TBST) for 2 h at room temperature. Membranes were incubated overnight at 4 °C with primary antibodies. Subsequently, membranes were incubated with the appropriate horseradish peroxidase-conjugated specific goat anti-rabbit secondary antibody (Affinity Biosciences; Cincinnati, USA) for 2 h at room temperature and then washed with TBST three times. Enhanced chemiluminescence chromogenic substrate (Millipore; Billerica, USA) was used to visualize the bands and the intensity of the bands was quantified by Image Lab software (Bio-Rad, CA, USA).

#### Cell proliferation assays

Cell proliferation was assessed by using Cell Counting Kit-8 (CCK8) (Bimake; Houston, USA) according to the manufacturer’s instruction. Briefly, the cells were seeded in 96-well plates at a density of 8 × 10^3^ cells per well. After 48 h of culture, cells were treated with 10 μL CCK8 kit to assess the proliferation potential. The cells were incubated at 37 °C for another 2 h and then read at 450 nm with Synergy ™ H1 microplate reader (BioTek Instruments Inc., Winooski, USA). All experiments were performed in triplicate.

#### IncuCyte live cell imaging system

Imaging was performed using the Incucyte Zoom Live-Cell Imaging System from Essen Bioscience (Ann Arbor, MI, USA). Cell confluence (%) was calculated using Incucyte Zoom software by phase-contrast images. Cells were scanned every 4 h from 0 to 96 h after treatment.

### Statistical analysis

Results were presented as means ± standard error (SE) of the mean for at least three independent biological replicates. Two-tailed Student’s t-tests were used for continuous variables. Kaplan–Meier analysis and log-rank test were used to evaluate the differences in patient’s survival. For statistical correlation, Spearman correlation coefficient was used according to requirements. Statistical analyses were performed utilizing the statistical software in GraphPad Prism version 8.0. Data were considered statistically significant as follows: * represents P < 0.05; ** represents P < 0.01; and *** represents P < 0.001.

## Results

### Genetic alterations in m6A regulators are widespread in pancreatic cancer

**Fig. 1:**
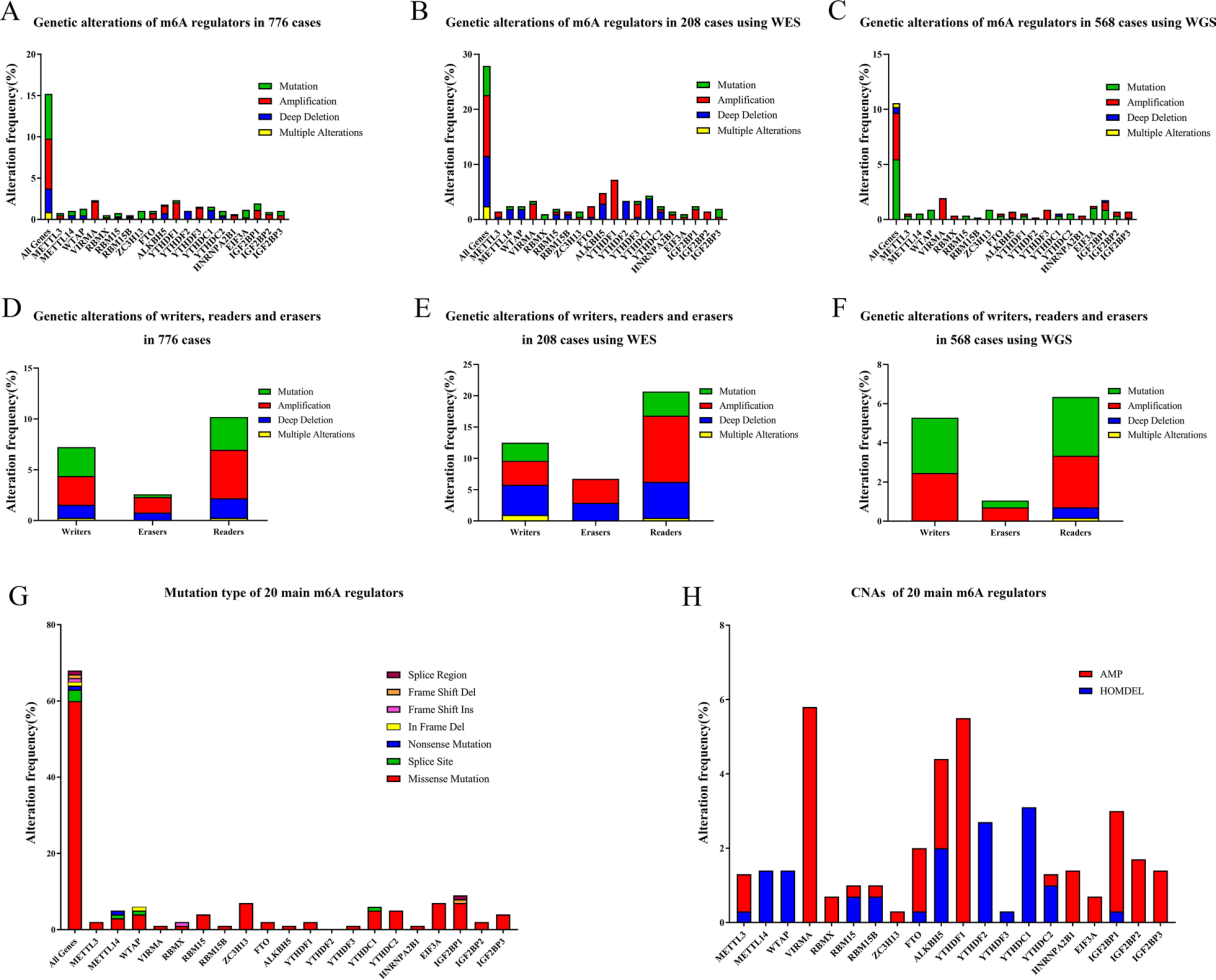
Genetic alterations of N6-methyladenosine (m6A) regulators in pancreatic cancer. **A** Genetic alterations of 20 main m6A regulators, including 8 writers, 2 erasers, and 10 readers, in 776 cases from 4 pancreatic cancer studies. **B** Genetic alterations of m6A regulators in 208 cases using whole exome sequencing (WES). **C** Genetic alterations of m6A regulators in 568 cases using whole genome sequencing (WGS). **D** Genetic alterations of m6A writers, erasers, and readers from 776 cases. **E** Genetic alterations of m6A writers, erasers, and readers from 208 cases using WES. **F** Genetic alterations of m6A writers, erasers, and readers from 568 cases using WGS. **G** Mutation types present in 20 main m6A regulators from 776 cases. **H** Copy number alterations (CNAs) in 20 main m6A regulators from 776 cases

m6A methylation is dynamically mediated by m6A “writers” and “erasers,” and is recognized by “readers.” We initially used cBioPortal to evaluate genetic alterations in 20 main m6A regulators, including 8 writers, 2 erasers, and 10 readers. This was conducted in 776 cases from 4 pancreatic cancer studies, including Pancreatic Cancer (UTSW, Nat Commun 2015) [5], Pancreatic Adenocarcinoma (TCGA, Firehose Legacy), Pancreatic Adenocarcinoma (QCMG, Nature 2016) [22], and Pancreatic Adenocarcinoma (ICGC, Nature 2012) [23] (Fig. 1). We found that the total alteration frequency of these 4 studies was 15.21% (118/776). Among them, the alteration frequencies of amplification, mutation, and deep deletion were 6.06% (47/776), 5.41% (42/776) and 2.84% (22/776), respectively. Only 0.90% (7/776) of these cases had two or more alterations (Fig. 1A). However, among these 4 studies, Pancreatic Cancer (UTSW, Nat Commun 2015) and Pancreatic Adenocarcinoma (ICGC, Nature 2012) used whole exome sequencing (WES); Pancreatic Adenocarcinoma (TCGA, Firehose Legacy) and Pancreatic Adenocarcinoma (QCMG,Nature 2016) used whole genome sequencing (WGS). So we have divided the cases and identified the alteration frequency in WGS and WES cases respectively. The results showed that the total alteration frequency of 208 cases using WES was 27.88% (58/208). Among them, the alteration frequencies of amplification, mutation, and deep deletion were 5.29% (11/208), 11.06% (23/208) and 9.13% (19/208), respectively. Only 2.40% (5/208) of these cases had two or more alterations (Fig. 1B). In 568 cases using WGS the total alteration frequency of was 10.56% (60/568). Among them, the alteration frequencies of amplification, mutation, and deep deletion were 5.46% (31/568), 4.23% (24/568) and 0.51% (3/568), respectively. Only 0.35% (2/568) of these cases had two or more alterations (Fig. 1C). Thus, amplification was the most common type of genetic alteration among the 20 main m6A regulators in pancreatic cancer. The overall average alteration frequencies of m6A writers, erasers, and readers were 1.03%, 1.42%, and 1.31%, respectively (Fig. 1D). The alteration frequencies of m6A writers, erasers, and readers in WGS and WES cases were shown in Fig. 1E, F respectively. Moreover, m6A writers VIRMA (2.32%) and WTAP (1.29%), m6A eraser ALKBH5 (1.80%), and m6A readers YTHDF1 (2.32%), IGF2BP1 (1.93%), YTHDF3 (1.55%), and YTHDC1 (1.55%) showed higher alteration frequencies. The total alteration frequencies of Pancreatic Cancer (UTSW, Nat Commun 2015), Pancreatic Adenocarcinoma (TCGA, Firehose Legacy), Pancreatic Adenocarcinoma (QCMG, Nature 2016), and Pancreatic Adenocarcinoma (ICGC, Nature 2012) were 50.46% (55/109), 21.62% (40/185), 5.22% (20/383), and 3.03% (3/99), respectively. Pancreatic Adenocarcinoma (QCMG, Nature 2016), and Pancreatic Adenocarcinoma (ICGC, Nature 2012) only had mutation alterations (Additional file 2).

**Fig. 2:**
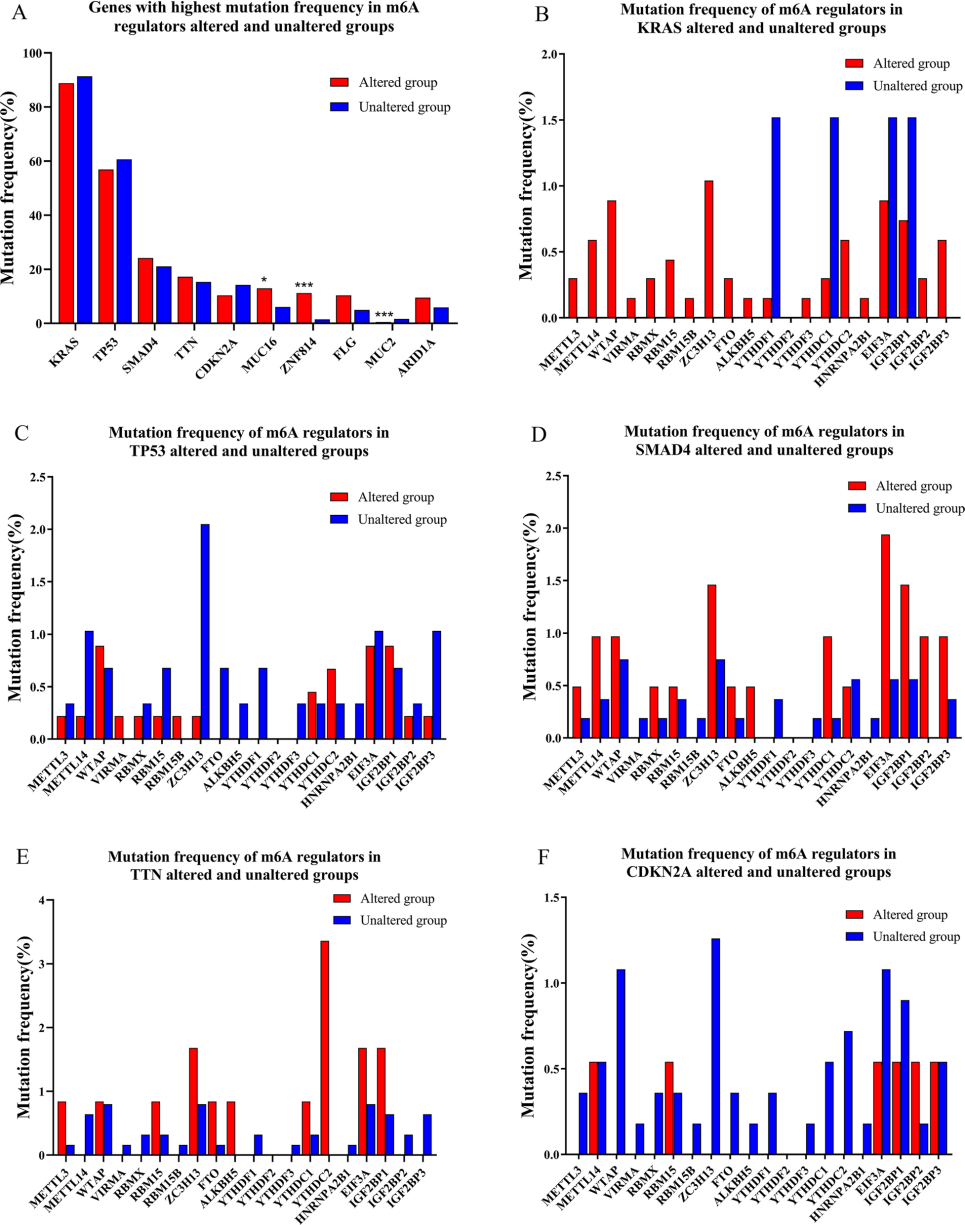
Mutation alterations of 20 main m6A regulators in pancreatic cancer. **A** Genes with highest mutation frequency in m6A regulators altered and unaltered groups. **B**–**F** Mutation frequencies of 20 main m6A regulators in unaltered and altered KRAS, TP53, SMAD4, TTN and CDKN2A groups. Red represents an altered group, and blue represents an unaltered group

There were 741 cases with mutation alterations among the 4 studies. The top five genes with the highest mutation frequency were KRAS, TP53, SMAD4, TTN, and CDKN2A. Their mutation frequencies were 91.00%, 60.10%, 21.60%, 15.50%, and 13.60%, respectively. SMAD4 and TTN showed higher mutation frequency in m6A regulators altered group, while KRAS, TP53 and CDKN2A showed lower mutation frequency in m6A regulators altered group. Though, there was no statistically significant difference between them (Fig. 2A). Next, we analyzed the mutation frequencies of 20 main m6A regulators in the altered and unaltered groups of these five genes. We found that YTHDF2 did not mutate in any group. Except for YTHDF1, YTHDC1, EIF3A and IGF2BP1, the mutation frequencies of other m6A regulators were higher in the altered KRAS group than unaltered group. The mutation frequencies of most m6A regulators were higher in the altered SMAD4 group than unaltered group, except for VIRMA, RBM15B, YTHDF1, YTHDF3, YTHDC2 and HNRNPA2B1. The mutation frequencies of METTL3, WTAP, RBM15, ZC3H13, FTO, ALKBH5, YTHDC1, YTHDC2, EIF3A and IGF2BP1 were higher in the altered TTN group than unaltered group; while the other m6A regulators showed lower mutation frequencies in the altered TTN group. Except for WTAP, VIRMA, RBM15B, YTHDC1, YTHDC2 and IGF2BP1, the mutation frequencies of other m6A regulators were lower in the altered TP53 group than unaltered group. The mutation frequencies of most m6A regulators were lower in the altered CDKN2A group than unaltered group, except for RBM15 and IGF2BP2. Details were shown in Fig. 2B–F and Additional file 3. We then collected the mutation data for 20 main m6A regulators across these 4 studies. We found that there were 68 mutations in these m6A regulators in all cases. The most frequent mutations were missense mutations (60 missense mutations, 3 splice sites, 1 nonsense mutation, 1 in frame deletion, 1 frame shift ins, 1 frame shift deletion, and 1 splice region). The number of cases with missense mutations were as follows: seven in ZC3H13, EIF3A, and IGF2BP1; five in YTHDC1 and YTHDC2; four in WTAP, RBM15, and IGF2BP3; three in METTL14; two in METTL3, FTO, YTHDF1, and IGF2BP2; and 1 in VIRMA, RBMX, RBM15B, ALKBH5, YTHDF3, and HNRNPA2B1 (Fig. 1G). The overall average number of mutations in m6A writers, erasers, and readers was 3.5, 1.5, and 3.7, respectively. Among these three, the readers showed the highest mutation frequencies. Moreover, IGF2BP1 showed the largest overall number of cases with mutations (7 missense mutations, 1 frame shift deletion, and 1 splice region, Fig. 1G).

**Fig. 3:**
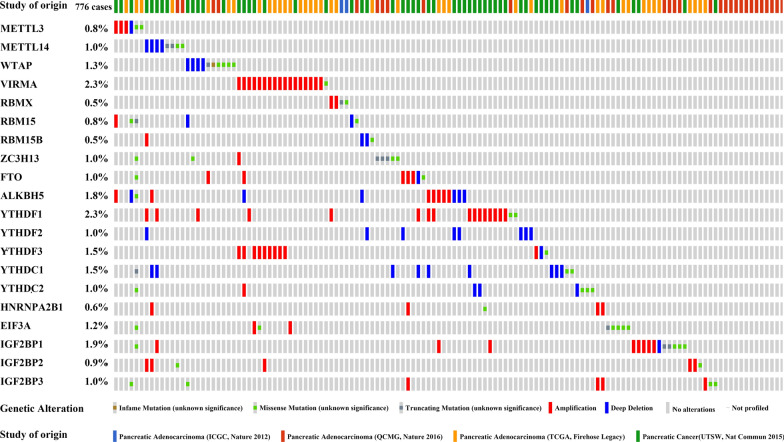
The tab OncoPrint showed an overview of genetic alterations of m6A regulators in pancreatic cancer. Only the proportion of cohorts containing the genomic alterations was shown in this figure. Each column was for individual case

We investigated the copy number alteration data across these 4 studies, and found that there were 293 cases with CNAs. VIRMA, YTHDF1, IGF2BP2, IGF2BP3, HNRNPA2B1, RBMX, EIF3A, and ZC3H13 only demonstrated amplified (AMP) alterations, and the alteration frequencies were 5.80%, 5.50%, 1.70%, 1.40%, 1.40%, 0.70%, 0.70%, and 0.30%, respectively. YTHDC1, YTHDF2, METTL14, WTAP, and YTHDF3 had only homozygously deleted (HOMDEL) alterations, and the alteration frequencies were 3.10%, 2.70%, 1.40%, 1.40%, and 0.30%, respectively. METTL3, RBM15, RBM15B, ALKBH5, FTO, IGF2BP1, and YTHDC2 showed both AMP and HOMDEL alterations, and the alteration frequencies were 1.00% and 0.30%, 0.30% and 0.70%, 0.30% and 0.70%, 2.40% and 2.00%, 1.70% and 0.30%, 2.70% and 0.30%, and 0.30% and 1.00%, respectively (Fig. 1H). The specific genetic alterations of the 776 cases were shown in Fig. 3.

### Transcription levels of m6A regulators in pancreatic cancer

**Fig. 4:**
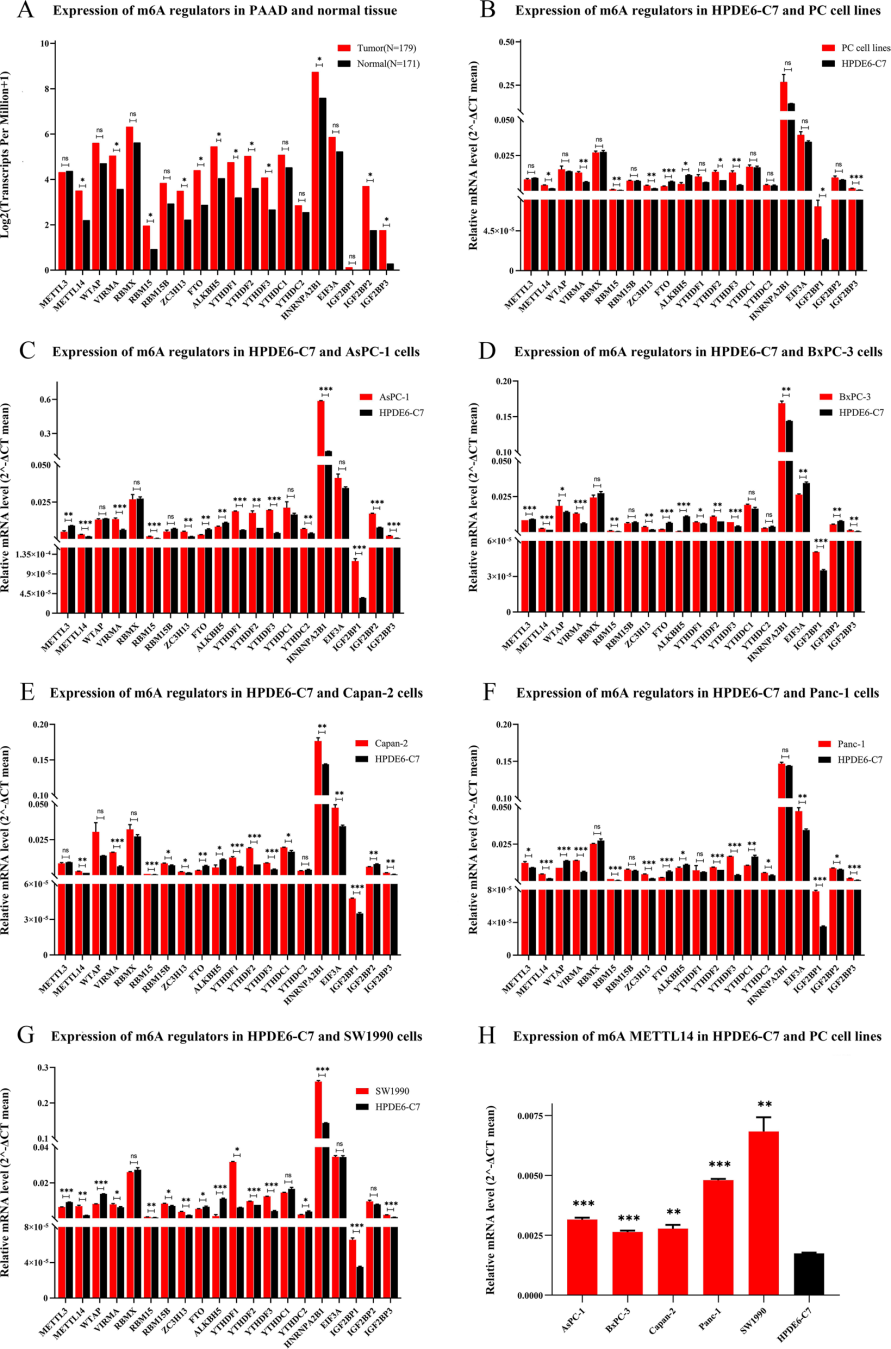
Transcription levels of 20 main m6A regulators in pancreatic cancer. **A** mRNA expression of 20 main m6A regulators in 178 pancreatic adenocarcinoma (PAAD) and 171 normal samples (GEPIA). Red represents tumor tissue, and black represents normal tissue. **B**–**G** RT-qPCR results of m6A regulators in human pancreatic cancer (PC) cell lines (AsPC-1, BxPC-3, Capan-2, Panc-1, and SW1990) and the control cell line HPDE6-C7. **H** METTL14 mRNA expression significantly up-regulated in all the five PC cell lines. Data were represented as the mean ± SE. ns represents not significant; * represents P < 0.05; ** represents P < 0.01; and *** represents P < 0.001

We sought to determine whether or not genetic alterations affect the expression of m6A regulators in pancreatic cancer. We explored the mRNA expression of m6A regulators between PAAD and normal samples by using the GEPIA. We found that the mRNA expression levels of almost all m6A regulators were higher in PAAD samples except for METTL3. Moreover, the expression differences of METTL14, VIRMA, RBM15, ZC3H13, FTO, ALKBH5, YTHDF1, YTHDF2, YTHDF3, HNRNPA2B1, IGF2BP2 and IGF2BP3 between tumor and normal tissues were statistically significant (Fig. 4A). Among them, IGF2BP3 showed the greatest difference in mRNA expression between PAAD patients and healthy individuals (Additional files 4A, 5). Further, we used the UALCAN to analyze the relationship between IGF2BP3 expression and multiple clinicopathological characteristics of 178 PAAD samples and 4 normal samples in TCGA. The results showed that this difference was not associated with tumor grade, but was closely related to disease stages, lymph node metastasis, race, gender, age, drinking habits, diabetes status, and pancreatitis status. Although only stage 2 PAAD patients showed statistically significant differences in IGF2BP3 expression compared with healthy individuals, the median IGF2BP3 expression was higher in stage 2 to 4 patients than in healthy individuals. This may be due to the other groups having insufficient samples. There was almost no statistically significant difference in IGF2BP3 expression in PAAD patient samples among different tumor grades, disease stages, lymph node metastasis, race, gender, age, drinking habits, diabetes status, and pancreatitis status (Additional files 4B-I, 6).

To verify the abnormal expression of m6A regulators in pancreatic cancer, we performed RT-qPCR in five human PC cell lines (AsPC-1, BxPC-3, Capan-2, Panc-1, and SW1990) and the control cell line HPDE6-C7. The results showed that the mRNA expression levels of most m6A regulators were higher in PC cell lines except for METTL3, RBMX, RBM15B, FTO and ALKBH5. Moreover, the expression differences of METTL14, VIRMA, RBM15, ZC3H13, FTO, ALKBH5, YTHDF2, YTHDF3, IGF2BP1 and IGF2BP3 between PC cell lines and HPDE6-C7 cell were statistically significant (Fig. 4B–G). Among m6A writers and erasers, the expression of METTL14 showed the greatest difference between PC cells and HPDE6-C7 cell. Compared with HPDE6-C7 cell, the mRNA expression of METTL14 in all the five PC cell lines were significantly up-regulated (Fig. 4H).

**Fig. 5:**
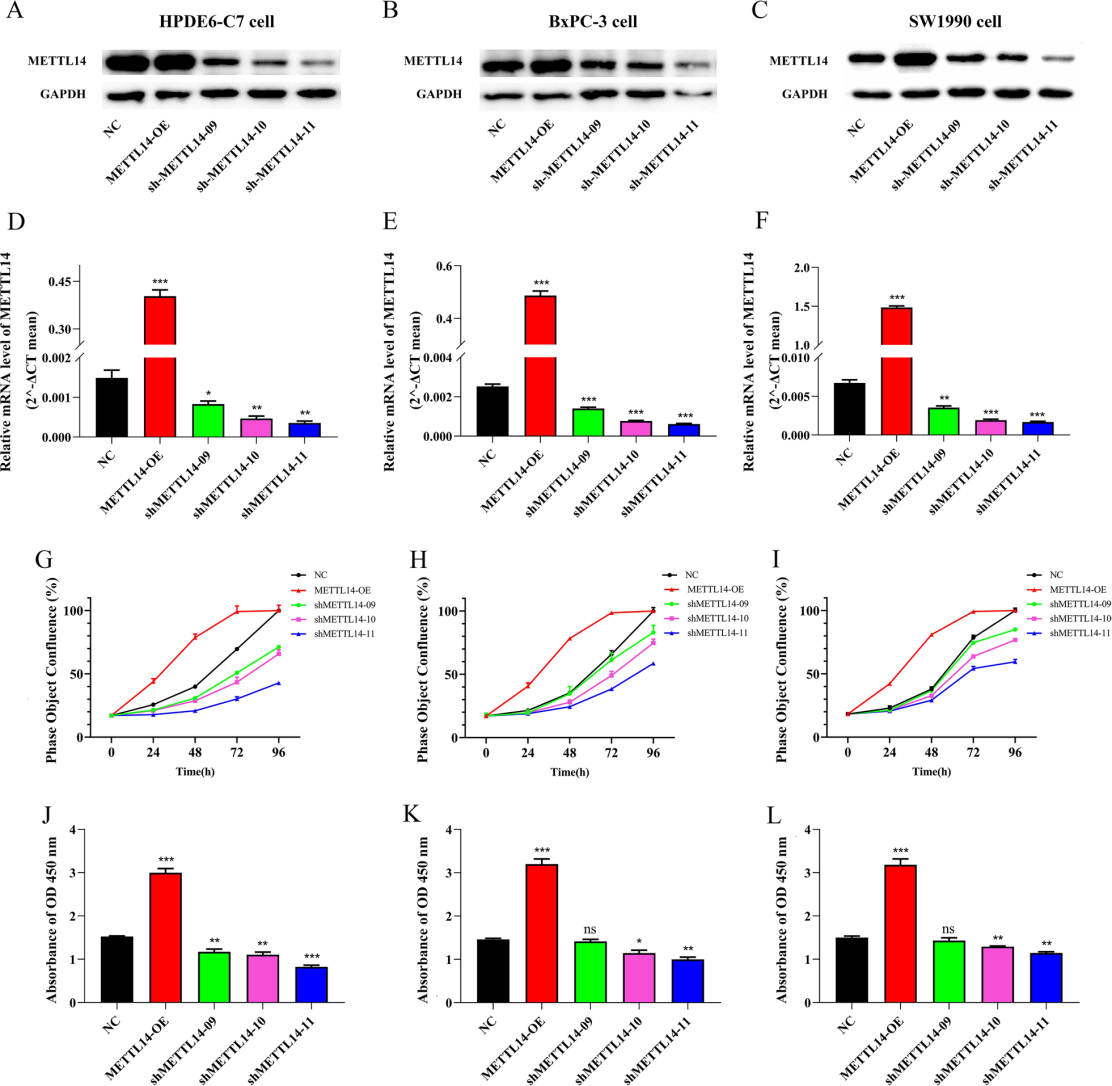
Validation of the function of METTL14 in tumor proliferation. **A**–**F** Real-time quantitative polymerase chain reaction (RT-qPCR) and western blotting (WB) results of METTL14 knockdown and overexpression in PC cell lines (BxPC-3 and SW1990) and HPDE6-C7 cell. **G**–**I** IncuCyte live cell imaging system results of growth curve in BxPC-3, SW1990 and HPDE6-C7 cells transfected with control, shRNAs and overexpression plasmids (0–96 h after treatment). **J**–**L** CCK8 analysis of BxPC-3, SW1990 and HPDE6-C7 cells transfected with control, shRNAs and overexpression plasmids (48 h after treatment). NC represents negative control group; shMETTL14-09, shMETTL14-10, and shMETTL14-11 represent cells transfected with corresponding shRNAs groups; METTL14-OE represents cells transfected with METTL14 overexpression plasmid group; ns represents not significant; * represents P < 0.05; ** represents P < 0.01; and *** represents P < 0.001

To identify the function of METTL14 in tumor proliferation, we knocked down METTL14 in PC cell lines (BxPC-3 and SW1990) and HPDE6-C7 cell using shRNAs (shMETTL14-09, shMETTL14-10, and shMETTL14-11). METTL14 knockdown was confirmed by RT-qPCR and Western blotting (Fig. 5A–F). ShMETTL14-11 suppressed the expression of METTL14 most. IncuCyte live cell imaging system results showed that knockdown of METTL14 significantly suppressed cell proliferation in BxPC-3, SW1990 and HPDE6-C7 cells. Similar results of CCK8 (48 h after treatment) were observed in these cells, except for the differences between shMETTL14-09 and NC groups in BxPC-3 and SW1990 cells. Further, we transfected BxPC-3, SW1990 and HPDE6-C7 cells with METTL14-OE plasmid. Remarkably, IncuCyte live cell imaging system and CCK8 results both showed that METTL14 overexpression significantly increased the proliferation abilities of BxPC-3, SW1990 and HPDE6-C7 cells (Fig. 5G–L).

### Alterations in m6A regulators affect DFS/PFS and OS of pancreatic cancer patients

**Fig. 6:**
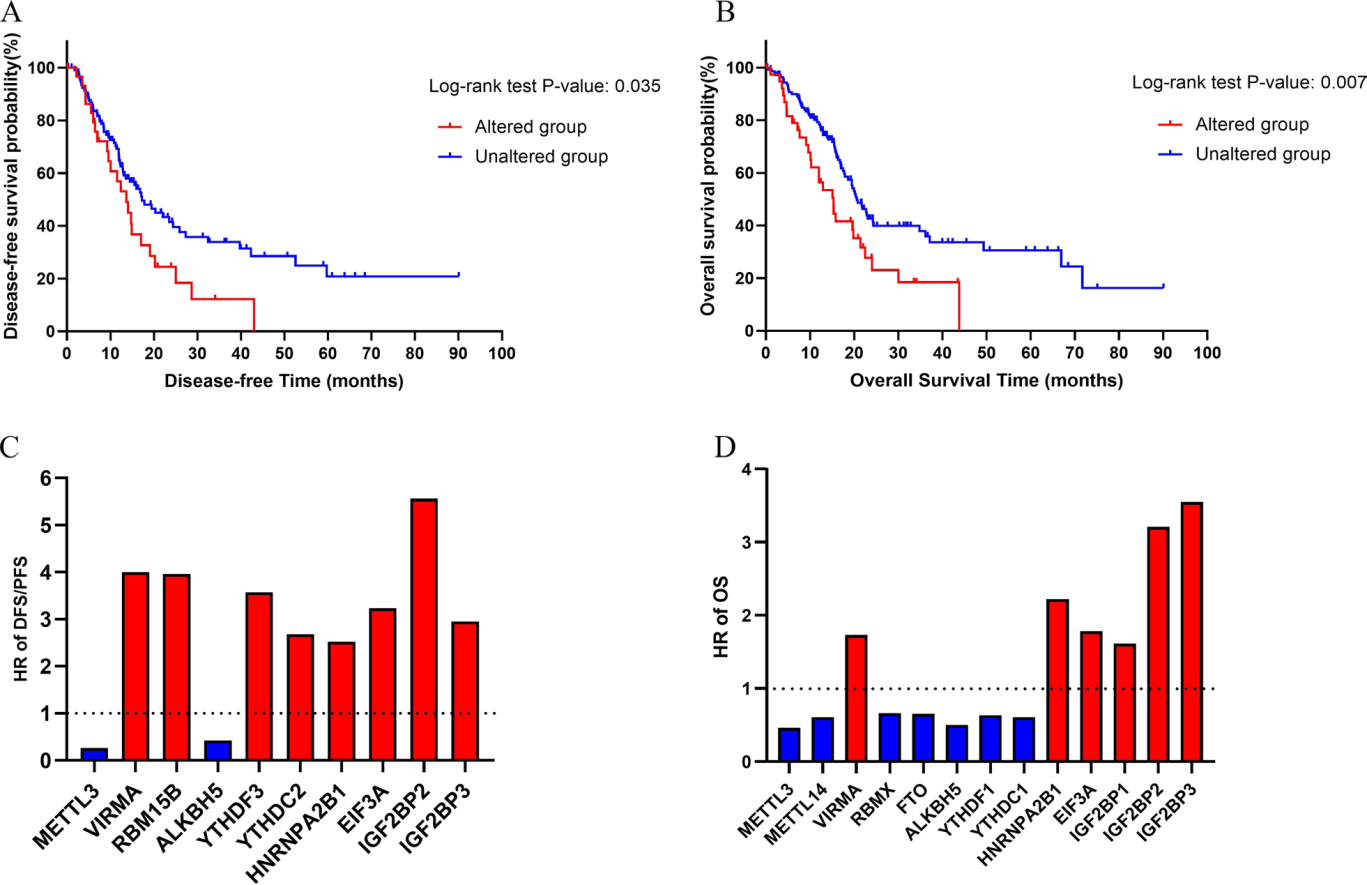
The survival data of pancreatic patients with or without genetic alterations in m6A regulators (cBioPortal). **A** Kaplan–Meier plots comparing the disease -free survival (DFS) in pancreatic cancer patients with or without genetic alterations of m6A regulators (log-rank test, P = 0.035). Genetic alterations in m6A regulators were associated with poorer DFS in pancreatic cancer patients. **B** Kaplan–Meier plots compared overall survival (OS) in pancreatic cancer patients with or without genetic alterations in m6A regulators (log-rank test, P = 0.007). Genetic alterations in m6A regulators were associated with poorer OS in pancreatic cancer patients. **C**, **D** A Kaplan–Meier plot and log-rank test analyzed the survival data of 177 patients with pancreatic cancer in the Cancer Genome Atlas (TCGA). We selected m6A regulators with genetic alterations showing statistically significant effects on pancreatic cancer patient survival (P < 0.05). Column bar graphs display the hazard ratio (HR) of DFS/PFS and OS of pancreatic cancer patients with genetic alterations in selected m6A regulators. Red represents HR > 1, and blue represents HR ≤ 1

To determine whether alterations in m6A regulators affected the DFS/PFS and OS of pancreatic cancer patients, we used the cBioPortal to evaluate the survival data of patients with or without genetic m6A regulator alterations from 4 pancreatic cancer studies. There were 30 cases with altered m6A regulators and 110 cases with unaltered m6A regulators in the DFS/PFS data. There were 39 cases with altered m6A regulators and 145 cases with unaltered m6A regulators in the OS data. Kaplan–Meier curve and log-rank test analyses revealed that genetic alterations in m6A regulators were associated with poorer DFS/PFS and OS in pancreatic cancer patients (log-rank test, P = 0.035 and 0.007, respectively, Fig. 6A, B, Additional file 7). We then analyzed the survival data of 177 pancreatic cancer patients in TCGA. After Kaplan–Meier plot and log-rank test analyses, we found that decreased METTL3 and ALKBH5 mRNA expression levels and increased VIRMA, RBM15B, YTHDF3, YTHDC2, HNRNPA2B1, EIF3A, IGF2BP2, and IGF2BP3 mRNA expression levels were significantly associated with poor DFS/PFS in pancreatic cancer patients (P < 0.05). Furthermore, pancreatic cancer patients with lower METTL3, METTL14, RBMX, FTO, ALKBH5, YTHDF1, and YTHDC1 mRNA expression levels or higher VIRMA, HNRNPA2B1, EIF3A, IGF2BP1, IGF2BP2, and IGF2BP3 mRNA expression levels had significantly poorer OS (P < 0.05, Fig. 6C, D). Interestingly, almost all m6A readers had a negative effect on the DFS/PFS and OS in pancreatic cancer patients, while m6A erasers had a positive effect. Most m6A writers simultaneously had a negative effect on the DFS/PFS, but a positive effect on the OS.

### Biological interaction network of m6A regulators in pancreatic cancer

**Fig. 7:**
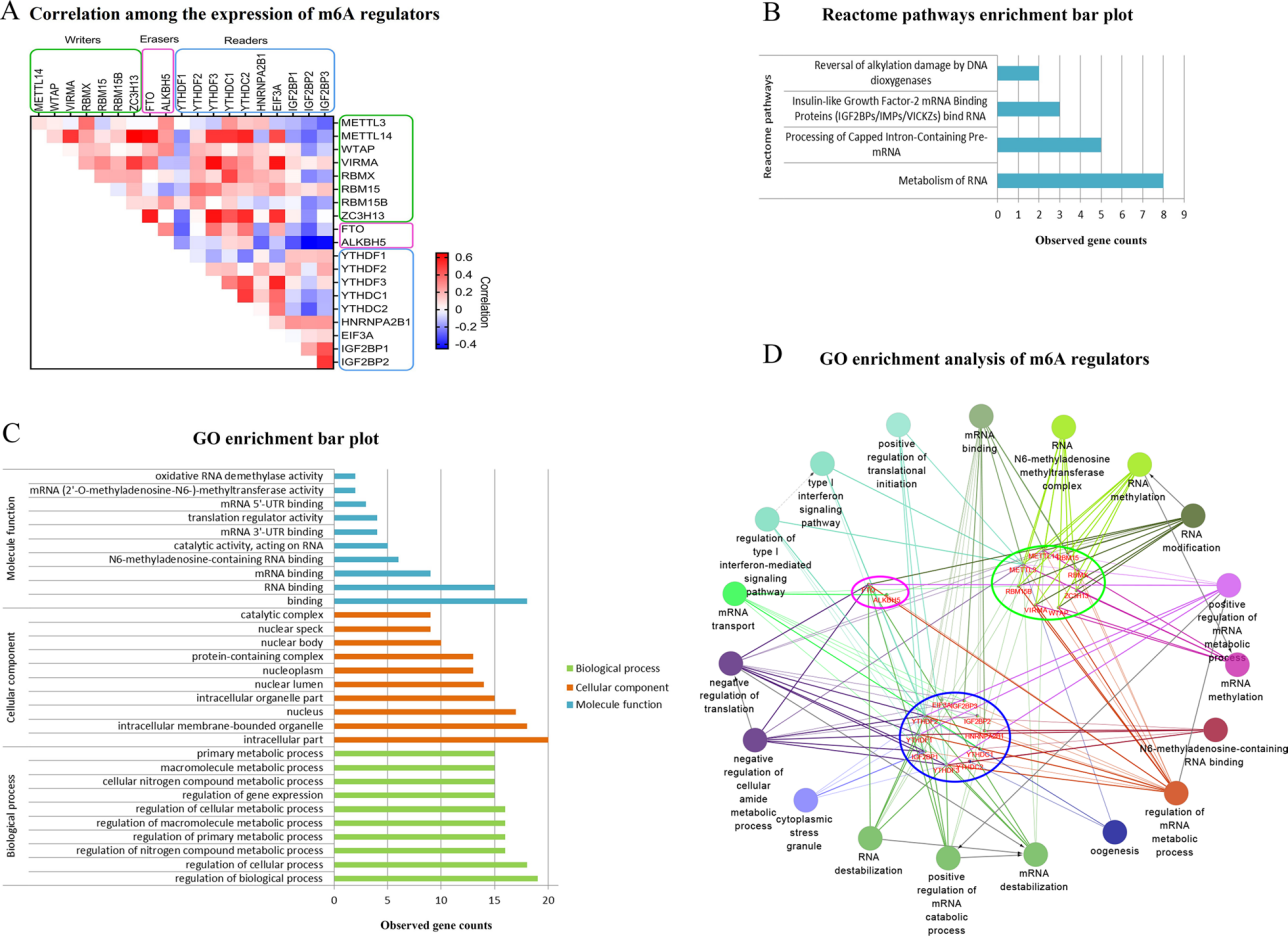
Biological interaction network of m6A regulators in pancreatic cancer. **A** Correlation heat map showing coexpression among m6A regulators in pancreatic cancer. Red represents a positive correlation between two genes, and blue represents a negative correlation. Darker colors indicate a greater correlation. **B**, **C** STRING was used to explore protein–protein interaction (PPI) networks between these writers, erasers, and readers in humans. **B** Significant Reactome pathway analysis shown via bar plots. The abscissa represents observed gene counts, and the ordinate represents the different pathways enriched. **C** Significant Gene Ontology (GO) term analysis is shown in bar plots. The abscissa represents observed gene counts, and the ordinate represents genes enriched in 3 hierarchical categories, including biological processes, cellular components, and molecular functions. **D** Cytoscape was used to visualize GO enrichment analysis of these 20 main m6A regulators in humans. m6A writers present in the green circle; m6A erasers present in the pink circle; and m6A readers present in the blue circle

Previous research has shown crosstalk among the m6A writers, readers, and erasers regulating cancer growth and progression [24]. We investigated whether this coexpression existed among m6A regulators in pancreatic cancer. We found that genes in the same functional category had highly correlated expression patterns. Moreover, the expression of most writers, erasers, and readers had a highly positive correlation. There were some exceptions to this observation. YTHDF1 expression was negatively correlated with the expression of most genes, except for a slight positive correlation with IGF2BP1/2/3. The expression of IGF2BP1/2/3, especially IGF2BP2 and IGF2BP3, was negatively correlated with the expression of most writers and erasers (Fig. 7A, Additional file 8). We used STRING to explore PPI networks between these writers, erasers, and readers in humans. The results show that EIF3A is expected from these networks, and the PPI enrichment P-value is less than 1.0e^−16^. Significant GO term analysis shows that m6A regulators are mainly located in the intracellular membrane-bounded organelles, nucleus, nuclear lumen, nucleoplasm, nuclear body, and catalytic complex. Here, the regulators participate primarily in N6-methyladenosine-containing RNA binding, mRNA 3′-UTR and 5′-UTR binding, catalytic activity on RNA, translation regulator activity, mRNA (2′-*O*-methyladenosine-*N*6-)-methyltransferase activity, and oxidative RNA demethylase activity. m6A regulators have an important role in the regulation of biological processes, including cellular process, nitrogen compound metabolic process, primary metabolic process, macromolecule metabolic process, cellular metabolic process, and gene expression. Reactome pathway enrichment analysis shows that these m6A regulators are enriched in the metabolism of RNA, carrying out processing of capped intron-containing pre-mRNA, insulin-like growth factor-2 mRNA binding proteins (IGF2BPs/IMPs/VICKZs) bind RNA, and reversal of alkylation damage by DNA dioxygenases (Fig. 7B, C, Additional file 9). In addition, we used Cytoscape to perform the crosstalk among m6A writers, readers, and erasers in humans, and visualized GO enrichment analysis of these 20 main m6A regulators in Fig. 7D. The results show that m6A readers participate in almost all the enriched pathways.

### Enrichment of coexpressed genes related to m6A regulators in pancreatic cancer

**Fig. 8:**
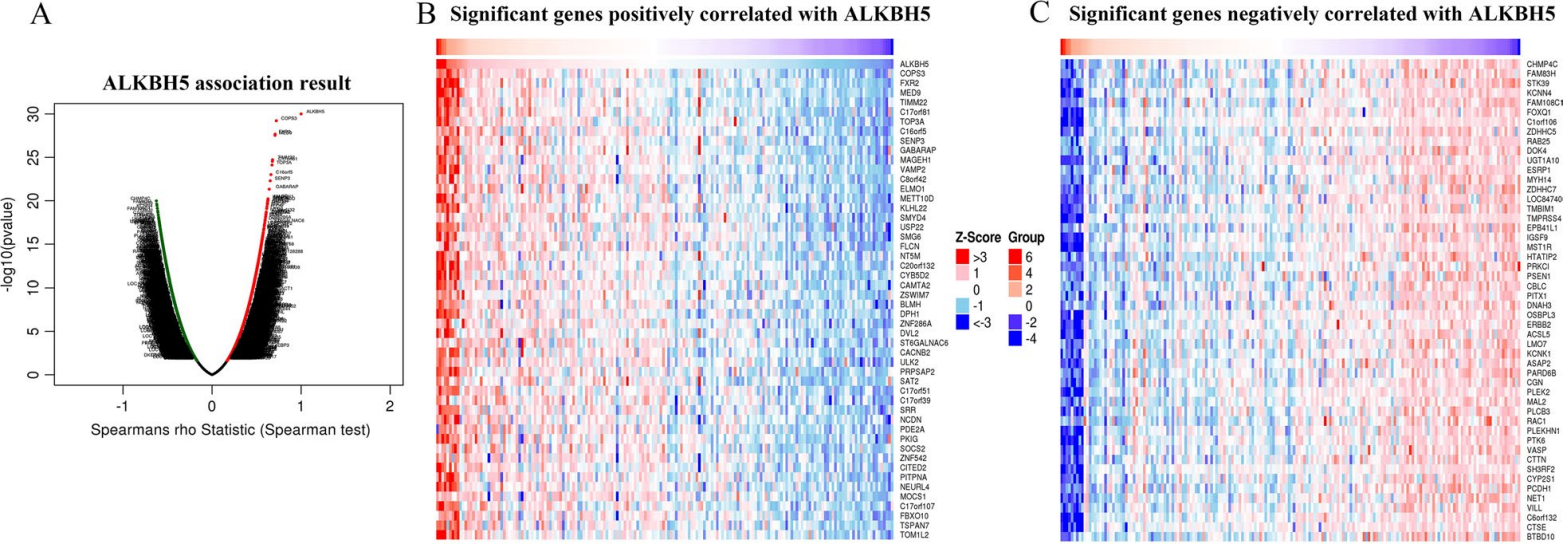
Genes differentially expressed in correlation with ALKBH5 in pancreatic cancer (LinkedOmics). **A** Spearman test was used to analyze correlations between ALKBH5 and genes differentially expressed in pancreatic cancer. **B**, **C** Heat maps showing the top 50 genes significantly positively and negatively correlated with ALKBH5 in pancreatic cancer. Red represents positively associated genes, and blue represents negatively associated genes

**Fig. 9:**
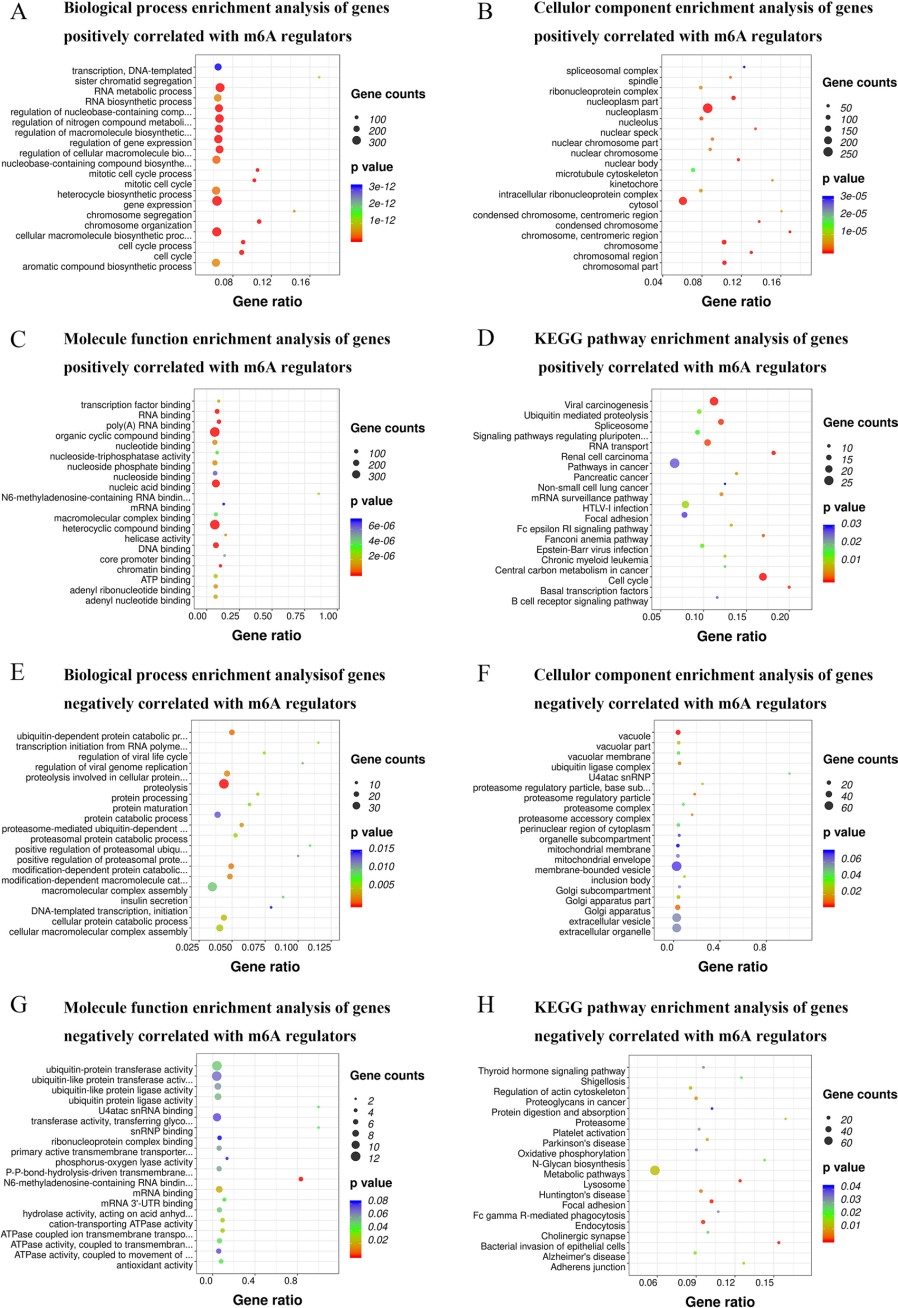
GO terms and KEGG pathways analysis of genes correlated with m6A regulators in pancreatic cancer. **A** Biological process enrichment analysis of genes positively correlated with m6A regulators. **B** Cellular component enrichment analysis of genes positively correlated with m6A regulators. **C** Molecular function enrichment analysis of genes positively correlated with m6A regulators. **D** KEGG pathways enrichment analysis of genes positively correlated with m6A regulators. **E** Biological process enrichment analysis of genes negatively correlated with m6A regulators. **F** Cellular component enrichment analysis of genes negatively correlated with m6A regulators. **G** Molecular function enrichment analysis of genes negatively correlated with m6A regulators. **H** KEGG pathways enrichment analysis of genes negatively correlated with m6A regulators

To investigate the coexpressed genes related to m6A regulators in pancreatic cancer, we used LinkedOmics to analyze the mRNA sequencing data of 20 main m6A regulators from 178 PAAD patients in TCGA (Additional file 10). For instance, the volcano plot (Fig. 8A) shows that 4102 genes (dark red dots) are significantly positively correlated with ALKBH5, whereas 2970 genes (dark green dots) show significant negative correlations (false discovery rate [FDR] < 0.01). The top 50 genes significantly positively and negatively correlated with ALKBH5 are shown in the heat map (Fig. 8B, C). We used DAVID to analyze significantly enriched GO terms of the top 50 genes positively or negatively correlated with m6A regulators. The results indicate that proteins encoded by genes significantly positively correlated with m6A regulators are localized mainly to the nucleoplasm, chromosome, nucleolus, microtubule cytoskeleton, ribonucleoprotein complex, spindle, and spliceosomal complex. The molecular functions of these genes are mainly regulation of RNA binding, poly (A) RNA binding, DNA binding, and organic cyclic compound binding. They are primarily involved in biological processes including RNA biosynthetic process, RNA metabolic process, transcription, DNA-templated transcription, protein modification process, and gene expression. KEGG pathways analysis shows enrichment in the cell cycle, spliceosome, RNA transport, mRNA surveillance pathway, ubiquitin mediated proteolysis, viral carcinogenesis, and cancer pathways. However, proteins encoded by genes significantly negatively correlated with m6A regulators are located mainly in the membrane-bound vesicles, vacuole, mitochondrial envelope, ubiquitin ligase complex, and Golgi apparatus. They mainly carry out molecular functions such as ubiquitin-protein transferase activity, transferase activity, transferring glycosyl groups, U4atac snRNA binding, snRNP binding, and *N*6-methyladenosine-containing RNA binding. These affect biological processes such as proteolysis, proteolysis involved in cellular protein catabolic process, ubiquitin-dependent protein catabolic process, and modification-dependent protein catabolic process. KEGG pathways analysis shows enrichment mainly in focal adhesion, lysosome, endocytosis, proteasome, protein digestion and absorption, and metabolic pathways (Fig. 9, Additional file 11, 12).

**Fig. 10:**
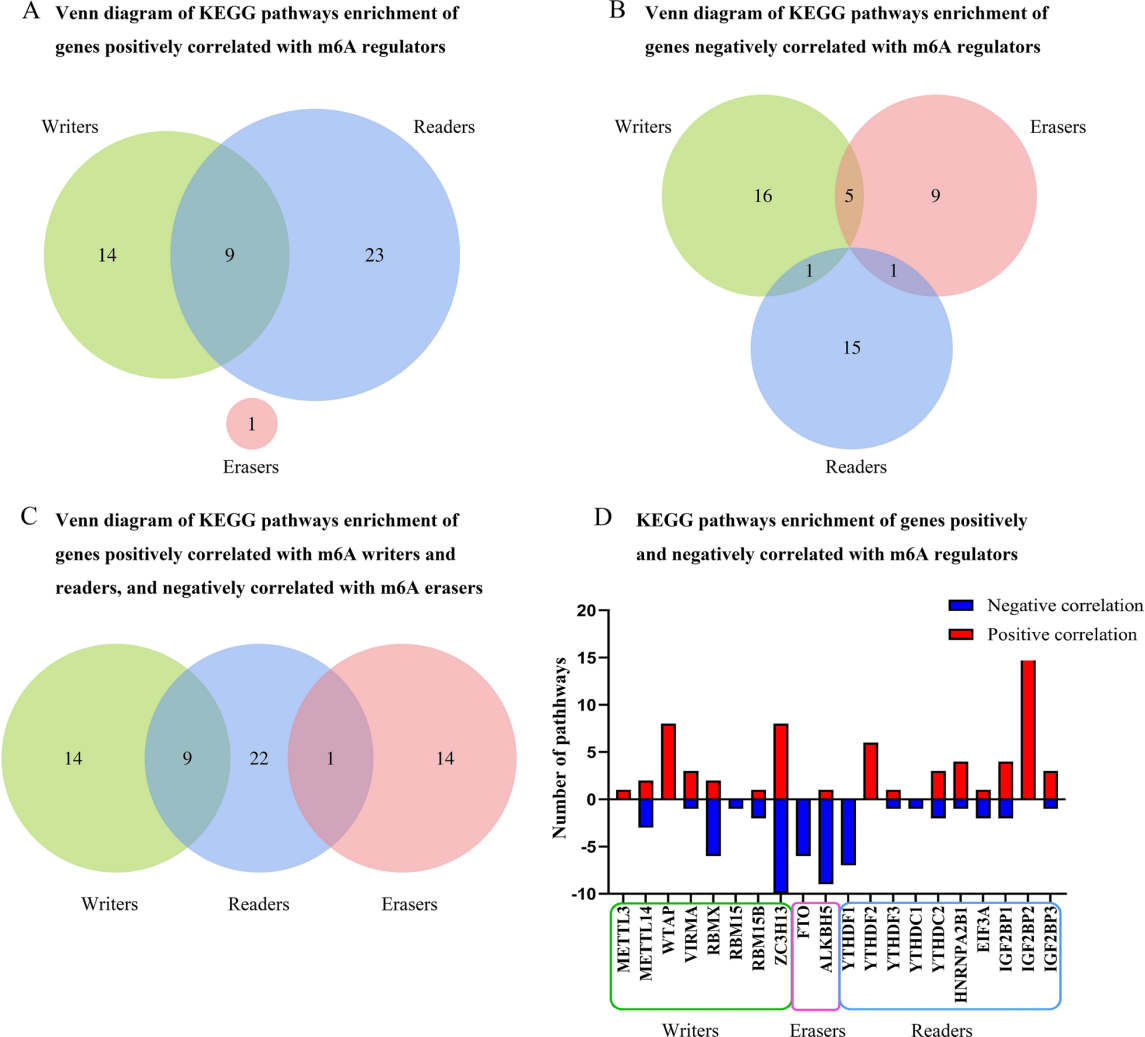
KEGG pathways enrichment analysis of genes correlated to m6A regulators. **A** Venn diagram of KEGG pathways enrichment analysis of genes positively correlated with m6A writers, erasers, and readers. **B** Venn diagram of KEGG pathways enrichment analysis of genes negatively correlated with m6A writers, erasers, and readers. **C** Venn diagram of KEGG pathways enrichment analysis of genes positively correlated with m6A writers and readers, and negatively correlated with m6A erasers. **D** KEGG pathways enrichment analysis of genes positively and negatively correlated with 20 main m6A regulators. Red represents positive correlation; and blue represents negative correlation

We used a Venn diagram to show that genes positively correlated with m6A writers were enriched to 23 pathways, readers were enriched to 32 pathways, and erasers were enriched to 1 pathway. There are 9 intersections between pathways enriched by genes positively correlated with m6A writers and readers, but there is no intersection between pathways enriched by genes positively correlated with erasers and writers, nor erasers and readers. Simultaneously, genes negatively correlated with m6A writers are enriched to 22 pathways, readers are enriched to 17 pathways, and erasers are enriched to 15 pathways. There are 5 intersections between pathways enriched by genes negatively correlated with m6A writers and erasers, 1 intersection between writers and readers, and 1 intersection between erasers and readers. Interestingly, there is 1 intersection between pathways enriched by genes positively correlated with m6A readers and genes negatively correlated with m6A erasers, but there is no intersection between pathways enriched by genes positively correlated with m6A writers and genes negatively correlated with m6A erasers (Fig. 10A–C, Additional file 13, 14, 15). KEGG pathways enrichment analysis of the top 50 genes significantly positively and negatively correlated with each m6A regulator reveals that m6A writers, erasers, and readers affect a number of signaling pathways in pancreatic cancer (Fig. 10D). These also have important roles in the occurrence and development of pancreatic cancer.

## Discussion

Over the past few years, with the development of biochemical and high-throughput sequencing technology, identifying the chemical structure and various functions of m6A RNA modification has become feasible. However, the underlying mechanisms of m6A RNA methylation in pancreatic cancer are still unknown. In this study, we therefore systematically investigated the molecular alterations and biological networks of 20 main m6A regulators in pancreatic cancer. Our results demonstrated that m6A RNA methylation regulators were prevalently subjected to genetic alterations, which are significantly correlated with m6A regulator expression and the survival prognosis of pancreatic cancer patients. Furthermore, we found that there was crosstalk among m6A writers, readers, and erasers in pancreatic cancer. Each of these m6A regulators plays a critical role in various downstream pathways, including cancer pathways.

Recently, growing evidence suggests that m6A RNA modification is involved in tumor proliferation, differentiation, tumorigenesis, invasion, and metastasis [25–27]. Emerging research also suggests that the differential expression and dysfunction of m6A regulators is involved in pancreatic cancer carcinogenesis and development. A recent study revealed that cigarette condensate promoted METTL3 to increase m6A modification via NF-κB associated protein, which resulted in excessive oncogenic microRNA-25 maturation in pancreatic cancer cells. Mature microRNA-25 and microRNA-25-3p inhibited PH domain leucine-rich repeat protein phosphatase 2, thereby activating the oncogenic AKT-p70S6K signaling pathway and promoting malignant phenotypes of pancreatic cancer cells [28]. It has been proved that ALKBH5 inhibited pancreatic cancer motility by demethylating lncRNA KCNK15-AS [29]. Dai et al. found that the m6A reader HNRNPA2B1 promoted the epithelial-mesenchymal transition in pancreatic cancer cells via the ERK/snail signaling pathway [30]. To obtain more detailed insight into the potential functions of m6A regulators in pancreatic cancer and its biological network, we conducted bioinformatics analysis of public sequencing data. We conducted this analysis to guide further studies of m6A RNA modification in pancreatic cancer and provide new strategies for pancreatic cancer treatment.

Previous research has revealed that there are widespread genetic alterations to m6A regulators across cancer types [13]. Our results confirmed that genetic alterations in m6A regulators were widespread in pancreatic cancer, and amplification was the most frequent alteration present. Our results showed KRAS, TP53, SMAD4, TTN and CDKN2A displayed the highest mutation frequencies in pancreatic cancer, which corroborate results of previous studies. We also found that m6A regulators were often mutated in groups with both altered KRAS and SMAD4 and unaltered TP53 and CDKN2A. Among the mutation alterations observed in 20 main m6A regulators, missense mutations occurred most frequently, and m6A readers showed relatively high mutation frequencies. Two hundred and ninety-three cases with CNAs were detected, and most m6A regulators were observed with CNAs. This indicates that genetic alterations in m6A regulators may occur frequently in pancreatic cancer, and the potential diagnostic and prognostic value of m6A regulators requires further clinical validation.

Genetic alterations often affect gene transcription and translation. A retrospective multi-cohort study showed that the m6A eraser ALKBH5 was an independent prognostic factor for pancreatic cancer, and its expression was positively associated with the OS of pancreatic cancer patients [31]. A multivariate Cox regression analysis indicated that nuclear WTAP expression was an independent prognostic indicator for pancreatic ductal adenocarcinoma, and high nuclear expression of WTAP was associated with a poor prognosis in pancreatic cancer patients [32]. Our study found that the mRNA expression levels of almost all m6A regulators were higher in PAAD samples except for METTL3. Furthermore, Kaplan–Meier curve and log-rank test analyses revealed that the median survival time of pancreatic cancer patients in m6A regulators altered group was significantly shorter than that of m6A regulators unaltered group (15.31 vs. 20.60 months, P = 0.035). Simultaneously, the median disease/progression-free survival of pancreatic cancer patients in m6A regulators altered group was also significantly shorter than that of m6A regulators unaltered group (13.67 vs. 17.28 months, P = 0.007). All these results indicated that m6A regulator alterations were associated with poorer DFS/PFS and OS in pancreatic cancer patients. In our opinion, genetic alterations appeared to affect the expression of m6A regulators and the survival prognosis of pancreatic cancer patients.

Previous study unveiled that the collaboration among m6A writers, erasers, and readers regulated tumor growth, angiogenesis, and progression [24]. Our correlation analysis revealed crosstalk among m6A writers, readers, and erasers in pancreatic cancer. We also found that genes in the same functional category showed highly correlated expression patterns. As the most prevalent epigenetic modification of eukaryotic mRNA and lncRNA, m6A is involved in every step of the RNA life cycle. Therefore, alterations in m6A regulators may cause changes in various downstream signaling pathways. We found that neighboring gene networks close to m6A regulators generally showed different degrees of alteration in pancreatic cancer. GO analysis of coexpression genes related to m6A regulators showed that they were mainly involved in RNA biosynthetic processes, RNA metabolic processes, transcription, DNA-templated transcription, protein modification processes, and gene expression. KEGG pathways analysis showed enrichment in the cell cycle, spliceosome, RNA transport, mRNA surveillance pathway, ubiquitin-mediated proteolysis, viral carcinogenesis, and cancer pathways. It is important to understand that m6A regulators interact with each other, and each of them affects various downstream pathways. Alteration even in only one m6A regulator can lead to dysregulation of gene expression, major dysfunction, or even cancer.

Studies have revealed that m6A RNA modification is involved in RNA transcription, splicing, nuclear export, localization, translation, and degradation [10]. With its effects on RNA processing, m6A participates in regulating gene expression, cellular processes, and influencing the occurrence and metastasis of various malignancies. Recent research has confirmed that ALKBH5 and FTO can demethylate m6A modification [33, 34], revealing that m6A modification is dynamically reversible. These results have greatly encouraged researchers to investigate the function of m6A and its regulatory mechanisms. However, the TCGA database has limitations. Firstly, there were too few samples in the normal group (the healthy control group) in this database, which may result in the comparison between the statistical significance of the normal and pancreatic cancer patient groups not reflecting the true situation. Secondly, the pancreatic cancer samples contained relatively few stage 3 and 4 patients. Nevertheless, the clinical reality is that more than 80% of pancreatic cancer patients present at a late stage at first diagnosis. Thirdly, pancreatic cancer samples were only from three ethnic groups. Most were Caucasian (156 cases), only 11 cases were Asian, and 6 cases were African–American. In fact, China has the highest morbidity and mortality for pancreatic cancer worldwide. Therefore, our results require further verification in clinical samples containing sufficient normal (healthy control) samples, advanced patients, and patients with different ethnicities. Moreover, some unexplainable problems are still present. For example, our results showed that most m6A writers had a negative impact on DFS/PFS, but also had a positive impact on the OS of pancreatic cancer patients. The molecular mechanisms of the biological functions of several regulators still require further experimental verification.

## Conclusions

This study has systematically analyzed molecular alterations in, and the functions of, 20 main mRNA regulators in pancreatic cancer. We ultimately determined that m6A regulators had widespread genetic alterations, and the expression levels of these regulators were significantly associated with the malignancy of pancreatic cancer. Furtherly, we found that there was a rich biological crosstalk among the m6A writers, readers, and erasers in pancreatic cancer, and alteration even in only one m6A regulator would lead to dysregulation of gene expression, major dysfunction, or even cancer. Moreover, m6A regulators were associated with the prognosis of pancreatic cancer patients. We believe that our results make a significant contribution to guide further studies of m6A RNA modification in pancreatic cancer and provide new strategies for pancreatic cancer treatment (Additional files 7, 8, 9, 10, 11, 12, 13, 14, 15).

## Supplementary Information

**Additional file 1.** Antibodies for WB and primers for RT-qPCR used in this study.

**Additional file 2.** Original data of genetic alterations of 20 main m6A regulators in 776 cases from 4 pancreatic cancer studies (cBioPortal). Mutation types and mutation number of different mutation types present in 20 main m6A regulators from 776 cases (cBioPortal). Copy number alterations (CNAs) in 20 main m6A regulators from 776 cases (cBioPortal). CNAs Copy number alterations, AMP Amplification, HOMDEL Homozygously deleted

**Additional file 3.** Genes with highest mutation frequency in m6A regulators altered and unaltered groups. Mutation frequencies of 20 main m6A regulators in unaltered and altered KRAS, TP53, SMAD4, TTN and CDKN2A groups (cBioPortal).

**Additional file 4.** The transcription levels of IGF2BP3 in pancreatic cancer. (A) The transcription levels of IGF2BP3 in 178 PAAD and 171 normal samples (GEPIA). (B-I) The relationship between IGF2BP3 expression and multiple clinicopathological characteristics of 178 PAAD samples and 4 normal samples in TCGA. These were stratified based on cancer stages, tumor grade, lymph node metastasis, race, gender, age, and other criteria (UALCAN). (B) Boxplot showing the relative IGF2BP3 expression in normal individuals and PAAD patients in stages 1, 2, 3, or 4. (C) Boxplot showing the relative IGF2BP3 expression in normal individuals and PAAD patients with grade 1, 2, 3, or 4 tumors. (D) Boxplot showing the relative IGF2BP3 expression in normal individuals and PAAD patients, with or without lymph node metastasis. (E) Boxplot showing the relative IGF2BP3 expression in normal individuals of any gender and male or female PAAD patients. (F) Boxplot showing the relative IGF2BP3 expression in normal individuals of any age and PAAD patients aged 21–40, 41–60, 61–80, or 81–100 years. (G) Boxplot showing the relative IGF2BP3 expression in normal individuals of any ethnicity and PAAD patients of Caucasian, African-American, or Asian ethnicity. (H) Boxplot showing the relative IGF2BP3 expression in normal individuals of any diabetes status and PAAD patients with or without diabetes. (I) Boxplot showing the relative IGF2BP3 expression in normal individuals of any pancreatitis status and PAAD patients with or without pancreatitis. Data were represented as the mean ± SE. ns represents not significant; * represents P < 0.05; ** represents P < 0.01; and *** represents P < 0.001.

**Additional file 5.** Transcription levels of 20 main m6A regulators between PAAD and normal samples (GEPIA). qRT-PCR results of m6A regulators in human PC cell lines (AsPC-1, BxPC-3, Capan-2, Panc-1, and SW1990) and the control cell line HPDE6-C7.

**Additional file 6.** UALCAN was used to explore statistical difference of IGF2BP3 transcription level between 178 cases of pancreatic adenocarcinoma and 4 normal samples in TCGA. These were stratified based on cancer stages, tumor grade, lymph node metastasis, race, gender, age, and other criteria (UALCAN). TCGA the Cancer Genome Atlas

**Additional file 7.** Original data of DFS and OS in pancreatic cancer patients with or without genetic alterations of 20 main m6A regulators from 776 cases (cBioPortal).

**Additional file 8.** Original data of correlation amongst m6A writers, readers, and erasers in pancreatic cancer(cBioPortal). We found that genes in the same functional category showed highly correlated expression patterns, and expression between most writers, erasers, and readers had a largely positive correlation.

**Additional file 9.** Original data of exploring protein–protein interaction networks between m6A writers, erasers, and readers in humans (STRING).

**Additional file 10.** LinkedOmics was used to explore genes positively or negatively correlated with 20 main m6A regulators in pancreatic cancer. The top 50 genes that were significantly positively and negatively correlated with m6A regulators were used to perform enrichment analyses of GO (BP, CC and MF) and KEGG pathways. GO Gene Ontology, CC Cellulor component, BP Biological process, MF Molecule function, KEGG Kyoto Encyclopedia of Genes and Genomes

**Additional file 11.** GO and KEGG pathways enrichment analysis of genes positively correlated with 20 main m6A regulators in pancreatic cancer (LinkedOmics).

**Additional file 12.** GO and KEGG pathways enrichment analysis of genes negatively correlated with 20 main m6A regulators in pancreatic cancer (LinkedOmics).

**Additional file 13.** The detail information of KEGG pathways enrichment analysis of genes positively correlated with m6A writers, erasers, and readers, respectively (LinkedOmics).

**Additional file 14.** The detail information of KEGG pathways enrichment analysis of genes negatively correlated with m6A writers, erasers, and readers, respectively (LinkedOmics).

**Additional file 15.** The detail information of KEGG pathways enrichment analysis of genes positively correlated with m6A writers and readers, and negatively correlated with m6A erasers (LinkedOmics).

## Data Availability

The data used and analyzed during the current study are available from TCGA and GTEx public dataset.

## Abbreviations

ALKBH5: Homolog AlkB family member 5
AMP: Amplified
CCK8 kit: Cell Counting Kit-8
CNAs: Copy number alterations
CT: Cycle threshold
DAVID: Database for annotation, visualization, and integrated discovery
DFS/PFS: Disease/progression-free survival
EIF3A: Eukaryotic translation initiation factor 3 subunit A
FBS: Fetal bovine serum
FDR: False discovery rate
FTO: Fat-mass-and obesity-associated protein
GEPIA: Gene Expression Profiling Interactive Analysis
GO: Gene Ontology
GTEx: Genotype-Tissue Expression
HNRNPA2B1: Heterogeneous nuclear ribonucleoproteins A2/B1
HOMDEL: Homozygously deleted
HR: Hazard ratio
IGF2BP: Insulin-like growth factor 2 mRNA-binding protein
KEGG: Kyoto Encyclopedia of Genes and Genomes
lncRNA: Long noncoding RNA
m6A: *N*6-Methyladenosine
METTL: Methyltransferase-like
METTL14-OE: METTL14 overexpression
mRNA: Messenger RNA
NC: Negative control
OS: Overall survival
PAAD: Pancreatic adenocarcinoma
PC: Pancreatic cancer
PPI: Protein–protein interaction
RBM15/15B: RNA-binding protein 15/15B
RT-qPCR: Real-time quantitative polymerase chain reaction
SE: Standard error
shRNAs: Short hairpin RNAs
SNPs: Single nucleotide polymorphisms
TBST: Tris-buffered saline-Tween 20
TCGA: The Cancer Genome Atlas
VIRMA: Vir like m6A methyltransferase associated protein
WB: Western blotting
WES: Whole exome sequencing
WGS: Whole genome sequencing
WTAP: Wilms tumor 1-associated protein
YTH: YT521-B homology
ZC3H13: Zinc finger CCCH domain-containing protein 13
