# Supplementary figures and images for: Genetic alterations and functional networks of m6A RNA methylation regulators in pancreatic cancer based on data mining
Source: J Transl Med. 2021 Jul 30;19:323. doi: 10.1186/s12967-021-03001-2 (PMC8325265; doi:10.1186/s12967-021-03001-2)

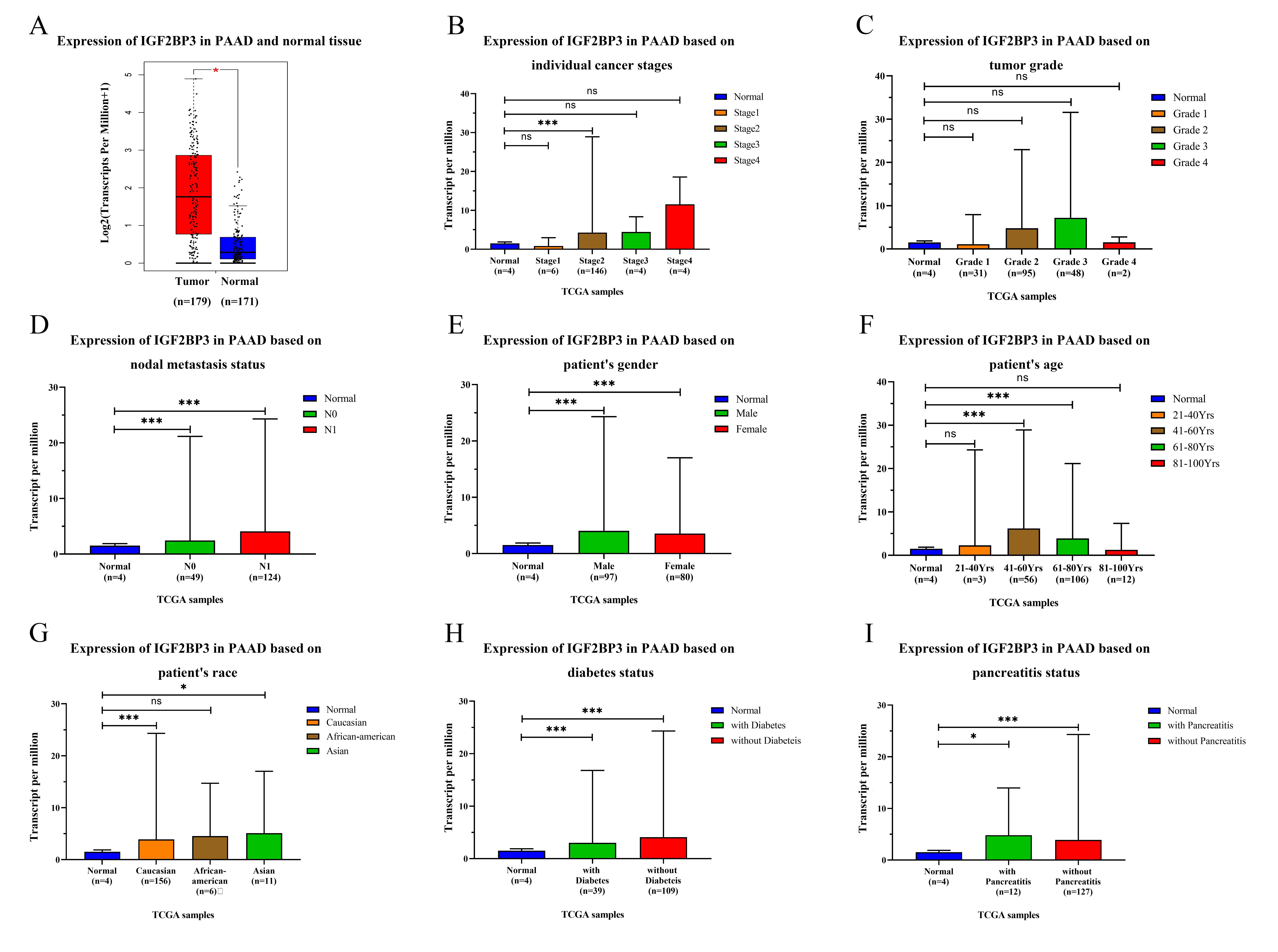

Supplement: Supplementary file 4 — Additional file 4. The transcription levels of IGF2BP3 in pancreatic cancer. (A) The transcription levels of IGF2BP3 in 178 PAAD and 171 normal samples (GEPIA). (B-I) The relationship between IGF2BP3 expression and multiple clinicopathological characteristics of 178 PAAD samples and 4 normal samples in TCGA. These were stratified based on cancer stages, tumor grade, lymph node metastasis, race, gender, age, and other criteria (UALCAN). (B) Boxplot showing the relative IGF2BP3 expression in normal individuals and PAAD patients in stages 1, 2, 3, or 4. (C) Boxplot showing the relative IGF2BP3 expression in normal individuals and PAAD patients with grade 1, 2, 3, or 4 tumors. (D) Boxplot showing the relative IGF2BP3 expression in normal individuals and PAAD patients, with or without lymph node metastasis. (E) Boxplot showing the relative IGF2BP3 expression in normal individuals of any gender and male or female PAAD patients. (F) Boxplot showing the relative IGF2BP3 expression in normal individuals of any age and PAAD patients aged 21–40, 41–60, 61–80, or 81–100 years. (G) Boxplot showing the relative IGF2BP3 expression in normal individuals of any ethnicity and PAAD patients of Caucasian, African-American, or Asian ethnicity. (H) Boxplot showing the relative IGF2BP3 expression in normal individuals of any diabetes status and PAAD patients with or without diabetes. (I) Boxplot showing the relative IGF2BP3 expression in normal individuals of any pancreatitis status and PAAD patients with or without pancreatitis. Data were represented as the mean ± SE. ns represents not significant; * represents P < 0.05; ** represents P < 0.01; and *** represents P < 0.001. [file 12967_2021_3001_MOESM4_ESM.tif]
